# Dr. Dwinelle, on Deep-Seated Dental Caries

**Published:** 1852-07

**Authors:** Wm. H. Dwinelle

**Affiliations:** Cazenovia, N. Y.


From the American Journal.

*On the Treatment of Deep-seated Dental Caries.* By Wm. H. Dwinelle, M. D., D. D. S., Cazenovia, N. Y.

From time immemorial it has been the highest aim of art to imitate nature. She has ever been the great ensample which we may continually approach, yet never reach; and those productions will ever be considered most perfect which most nearly resemble her own.

The sculptor who has produced the faultless form, is inferior to him who clothes his statue with a living sentiment, and makes the "marble to breathe"—yet man, the handiwork of the Divine, is infinitely superior to them both. The painter who gives you a likeness of your friend, is inferior to him who produces one, from which you feel thoughts are beating responsive to your own, and where her "rapt soul is sitting in her eyes." This is the highest attainment of art; and yet, how immeasurably do these impressions fall short of those of human magnetism and sympathy, which gush forth from the living, breathing presence.

In our own studio-laboratories, for the field of our profession is world-wide, and should embrace all art—the student who constructs a dental fixture to the satisfaction of his inexperienced patient, is greatly inferior to the master, who, in selection and arrangement, in form, and in color, closely studies the temperament and age, complexion and *physique*, of those to whom he would give the highest advantages of our art. And yet above all of these triumphs of skill, and they are great triumphs, nature still stands pre-eminent—above all art.

Again, the operator who restores the natural dental organs, and preserves them from destruction, has achieved vastly more than he who imitates and substitutes them ; but unless he has restored to them *all* of their functions, and preserved them in *all* of their vitality, he has fallen short of the high victory which awaited him.

The nearest assimilation to nature, is the highest point of perfection, and when we have secured the preservation of the teeth at the expense of their nerves, we cannot but concede that we are at least *one* remove, and we cannot estimate this, from that complete triumph, which would enable us to contrast a living with a dead organization. Though we have discovered much, and may still more, in regard to the character and functions of the nerves of the teeth, they may have a thousand hidden influences and uses we "wot not of." Here, then, is the highest assimilation to nature. Here, the loftiest triumph of art! She is restored and preserved in the exercise of *all* of her powers, whether they be of resistance to morbid influences from without, restorative upon herself, or reflective upon the whole system.

In view of the fact, of the great importance of reclaiming and securing teeth at the highest point of perfection, I propose to take into consideration an extreme class of teeth, which, until within a few years, have been suffered to perish, or have been indiscriminately removed. I refer to that class of teeth whose bone has been suffered to decompose until that portion immediately over and covering the nerve, has lost all, or nearly all of its component of lime, to remove which, would be to ensure the exposure of the nerve. I shall arrange this class of teeth under three distinct heads. Before, however, entering into a consideration of the three classes in their order, I will, by way of preparation, record a few aphorisms, or statements, in regard to the physiological construction, condition, and habits of the teeth.

Although the nerve of the tooth is manifestly the main channel of communication of nervous sensibility, and although no branches proceeding from the nerve itself are preceptible in the structure of the teeth, yet there is a subtle agency, which emanating from the nerve, ramifies through the tubuli of the teeth to the surface of the bone. At some future time, I propose to exemplify, in the most ample manner, the truth of the proposition, that the teeth have as legitimate a channel of nervous communication to their extremities, as any other highly endowed part of the body. It will be abundantly sufficient for my present purpose to state that I am fortified in my theory by several in our profession of the most extensive experience. Dr. Elisha Townsend, of Philadelphia, among others, who, in the April, 1851, No. of the Journal, page 260, says, "The question is often asked, why is the bone so tender and exquisitely painful as it often is, at the commencement of excavating, and yet sometimes entirely painless when the diseased portions of it are removed, why should it not be attributed to inflammation?— The teeth, though not highly organized, are supplied with a nerve in each fang, an artery and a vein; these give the internal vitality to the tooth, and pass that vitality through its whole bony structure, till they meet the enamel. If, then, the branches of the nerve pass through the whole bone of the tooth, to the investing surface called the enamel, it is easy to understand how the minute ends of these veins may be exquisitely sensitive when there is caries and inflammation.

"It is the experience of every one who has practiced dental surgery, that when he has cut into the pulp cavity, and severed the connection between the bony surface immediately over it, and the nerve and blood-vessels, the pain which was felt before in all parts of the tooth, immediately ceases, no portion of the cavity then being tender to the excavator, but the membrane or nerve. Does not this seem to prove, conclusively, the nerve to send minute microscopic branches through the whole tooth, which are the medium of sensation as long as the connection between them and the bone is not ruptured."

In many respects, the condition of the bone of the tooth, is strictly analogous to that of other parts of the system. When in a healthy condition, the bone of the teeth is not usually possessed of any great degree of sensitiveness, but when diseased or inflamed by exposure and contact with irritating substances, it becomes sensitive to the extreme. The gums, when healthy, can be cut with but comparatively little pain, but when inflamed, are exceedingly sensitive to the slightest touch. The eye can be almost rudely handled in its healthy state, without giving pain, and yet, in a few hours it may become so sensitive that a feeble ray of light will overpower it.

The condition of the teeth under some circumstances become highly exalted, so much so, that Niel and Magendie have ascribed to them the sense of taste.*

We can amputate diseased and intensely organized bone, and cast it aside in the same way we would an inflamed and sensitive fungus from healthy parts over which it reposed.

It seems to be a principle of physiology that the extremities of the nerves shall be endowed with the highest degree of sensitiveness. The severing of the median or sciatic nerves causes scarcely more pain than the excision of a few score of their myriad branches terminating on the surface; a severe scald, covering a considerable surface causes the most agonizing pain, and often, immediate death, whereas, the severing of every one of the principal nerves which gave vitality to the parts, would be attended with comparatively slight pain.

The most sensitive part of the healthy bone of a tooth, is at the boundary line between itself and the enamel, and most distant from the nerve—in fact, at the extreme *surface* of the bone. A patient will oftentimes experience far more pain while you are excavating parts where the bone loses itself in the enamel, than when you extirpate the nerve itself.

As the condition of the bone and nerves of the teeth are in many respects analogous to other parts of the system, so are they in most respects alike subject to medical and surgical remedial agencies. Palliatives, counter-irritants, blood-letting, blistering, anodynes, caustics, &c., &c., are all of them legitimate, and often successful remedies for restoring to healthful action diseased nerves which otherwise would have perished.

But to return to the classification of the order of teeth under consideration.

CLASS 1. Teeth, the nerves of which are only covered by

* Oliver's Physiology, p. 390, 3d ed.

softened bone, but which, otherwise, so far as can be ascertained, are healthy, or are capable of being restored to health.

Class 2. Teeth, whose nerves are by accident, in excavating or otherwise, exposed—even lacerated, but which are healthy, or are capable of restoration to health.

Class 3. Teeth, whose nerves are incapable of restoration, and whose destruction is inevitably required.

The first class of teeth I propose after necessary treatment, to fill without uncapping the nerve by removing the soft parts over it, with the reasonable expectation that I shall thereby permanently arrest the decay and secure to the tooth a full exercise of all its functions and powers.

The second class I propose to restore to health, if inflammation has not extended too far, then, after carefully capping the nerve, to fill the tooth in the usual manner, with a good prospect of securing its vitality, at least in a large majority of cases.

The third class I propose to treat by destroying and removing their nerves, and plugging them in the manner practiced by Maynard, Arthur, Dunning and others.

It will be readily conceded, that I have placed the most important class of teeth at the head of my list, for if they can be so treated as to entirely arrest their decay, and at the same time leave the nerve undisturbed to exercise its offices and powers without a prospect of their interruption, the operation must rank first in importance with any known in our profession.

For nearly ten years past, I have been in the practice of treating the class of teeth under consideration, in the manner referred to, and, too, with a degree of success which warrants me in speaking with considerable confidence on the subject.

In vol. 7, (1846,) page 74, of the American Journal of Dental Science, will be found an article "On the preparation of a cavity of a tooth preparatory to plugging," in which I advocated what was then considered a new and very unorthodox method of leaving decomposed bone immediately over the nerve, when its removal would expose it, and then proceed to fill it as usual. After giving a description of the extreme cases in which I felt justified in the operation, I proceeded as follows: "Under circumstances like the foregoing, after having thoroughly cleaned the walls and such parts of the bottom of the cavity of the tooth, as I can safely do, without interfering with the nerve, I wash out the cavity with a solution of soda, and then proceed to fill the tooth in the usual manner, taking care, however, especially if the parts are much softened immediately over the nerve, to skilfully build an arch over that point, so as to enable it to resist the nerve pressure of filling, finishing," &c. * * * * * *

"In considering teeth treated in the manner described, the question very naturally presents itself, '*does the decay go on?*' I answer emphatically, *No!* the decay is entirely a chemical action, and depends upon *external* agencies alone, for its progress, such as air and water, and such other influences as will promote a constant acidulated and decomposing action. The *decay* possesses no quality in itself of advancement, and when the cavity within the tooth is so completely and skilfully closed as to cut off all communication from *without*, it is as impossible for the decay to advance, as it would be if the nerve was capped with bone of the purest whiteness.

"The fact that the nerve is constantly receding with the age of the patient, to say the least, encourages our operation; and if our work is skilfully performed, and does not interfere with the internal membrane of the tooth, so as to cause inflammation justifies us in the conclusion that it is permanent and complete."

"Although the new doctrine was kindly received by most of the profession, it met with ridicule from others, accompanied by silly untruths and ridiculous hypotheses. In opposition to years of actual experience, one who disclaimed all personal knowledge or experience in my method of practice, vaguely remarks that the safety of the tooth, would be jeopardized by the partially decomposed bone, or "cartilaginous portion *drying down* and occupying less space than when the tooth was filled!"

Subsequently, in a letter to the Baltimore editor of the Journal, vol. 7, page 375, in defending my position, I attempted to prove that the bones are capable of great changes in their relative constituents, at times comparatively soft, and at others hard even to brittleness, and yet, through all their changes, retain their identity as bone. "A tooth may lose a fraction of its lime, and yet retain all of its essential qualities as a bone, as is manifest in the bones of different individuals, some of which almost vie with flint in texture, while others may be literally whittled with a knife. The bones of an infant contain but comparatively a small quantity of lime, and yet they possess all the essential qualities of bone—distinct living, organized bone. The bones of an aged person contain a much less quantity of gelatine, and yet they perform all of the requisite functions of bone."

Of the softened parts over the nerve, I said, "the decomposition, under the circumstances, will not go on, nor will it (the soft bone) *dry down*, and occupy less space than it did before, as cartilage would, situated in a *dry place*, and entirely remote from the influence of moisture. It would not dry down, I say especially as the tooth is not dead, and as the whole surrounding bone is of a porous quality, and the parts are immediately over a moist living nerve."

In allusion to the truthfulness of my proposition, and also to my desire thereby to benefit the profession, I concluded with "*tempus discernet!*" and time *has* shown!

Notwithstanding my subsequent experience had not only confirmed, but very extensively enlarged my views in the direction I had taken on this subject, still, with the exception of an occasional word of encouragement from some of my professional brethren, nothing came under my observation to induce me to think the profession, generally, would even sympathize with me in the great importance I had attached to the operation, until, as if to compensate for all of the past, suddenly and most unexpectedly I found the able pen of one of the best writers, as well as one of the best operators in our profession, advocating and defending, past all controversy, views and principles which heretofore for years, I had advocated and defended alone.

In No. III, of a series of articles on the "Treatment of dental caries, complicated with disorders of the pulp and peridental membrane, by Robert Arthur, D. D. S.," Journal of Dental Science, vol. 2, new series, page 1, the able author has in a far more graceful and concise manner than I could have done, expressed my views as well as practice, in treating that class of teeth whose nerves are only protected by a layer of decomposed, or partially decomposed, bone. His extensive experience, careful observation and correct judgment in this interesting field of inquiry, enables him to add much of great practical value to the profession. I think, however, as I can date the beginning of my experience in the peculiar mode of treatment under consideration, as far back as any one within my knowledge, and, as subsequent experience and observation would be likely to have a not unimportant bearing upon the subject, I shall be excused if I hastily describe my present method of treating the class of teeth under consideration, even though I may reiterate some things already expressed in the excellent article of Dr. A.

Indorsing anew, the principle laid down in my communication to the Journal in 1846, I repeat that the decay of the teeth is a chemical action, and depends upon external agencies for its progress; that this chemical operation is but the legitimate action of acid upon the tooth, uniting with its lime, which constitutes its base, gradually decomposing it, and leaving the gelatine, or animal matter, to waste by mechanical and other influences; and that this decomposing agency is generally derived from food and condiments retained in the interstices of or between, the teeth, or attached to some external nucleus until, if it is not already an acid, it is generated by acidous fermentation. That the decay possesses no quality in itself of advancement, &c., &c. In brief, when in the course of excavating a cavity preparatory to plugging, if, in my opinion, decomposition has so far proceeded as to reach the membrane of the nerve, and yet has not materially affected its healthful condition, and which said softened or decomposed bone, if removed, would ensure the exposure of the membrane or nerve, I invariably proceed to fill the tooth, leaving a portion of softened or decomposed bone, immediately over the nerve. I am particular to thoroughly excavate the walls and such parts of the bottom of the cavity of the tooth, as I can do with safety; and if any material pain continues in the tooth when I have ceased to excavate it, I dip a pledget of cotton in a concentrated solution of spirits of camphor, and apply it within the cavity, let it remain for a short time, then wash it out with a weak solution of soda dry it with soft paper and proceed as usual. If the soft parts are much sensitive to pressure, I either over-arch them with foil, or protect them with a concave cap of gold plate, so as to leave a slight space between it and the bone, immediately over the nerve. I often let this space be filled with some non-conductor, such as asbestos, liquid cuticle or wax.

I have pursued this course for several years past, and with almost universal success; it has been very rare, indeed, that the destruction of the pulp of the tooth has supervened, and only one instance has come to my knowledge where the loss of the tooth has resulted from the operation. My practice has embraced many hundred teeth treated in this manner, and has extended over a space of eight or ten years; and although I would naturally be as ridiculous to inquire into the after history of teeth treated in this manner, as circumstances would admit of, it is not to be presumed that I could have the opportunity of seeing, at the most, more than a majority of them.—Yet, from careful observation, I feel justified in saying, that I should be greatly disappointed, if in fifty cases treated in the manner described, more than one of them failed, and even in the case of failure, by careful subsequent attention and treatment, the tooth could be redeemed, though with the loss of its nerve.

Whatever of decomposing agencies that may be left in the softened parts, are either neutralized by treatment, or soon exhaust themselves.

Every avenue being cut off from all external influences, all further decomposition is arrested. Nature, aided by art, is left surrounded by the most favorable circumstances to recuperate herself. The *natural* condition and relation of all the parts, the invariable changes of time, the "common law" of physiology, all favor and promote the end.

The decomposed or softened bone over the nerve, after it is properly treated is the best possible covering for it; of itself, it is a non-conductor, and though wanting in vitality, nature does not regard it as extraneous. It adapts itself to the nerve as no artificial substance can, and finds that delicate boundary line, where the living and semi-vitalized organization, blends into and reposes unharmed upon the dead.

Repeated experiments have proven beyond doubt, that this very decomposed bone, which, by the way, is not always discolored, if protected by a proper filling, will uitimately receive any absorption, or deposit from the nerve a sufficient quantity of lime to compensate for its loss, and to render it as bony and compact as before. There are more reasons to believe that this effect may be produced artificially from without, I will refer to this on another page. Every one knows that the nerve cavities of our teeth are being constantly filled up and diminishsd by osseous deposit, that the nerves of the teeth are continually receding with our age, so that ultimately the nerve canal becomes entirely obliterated.

All of these circumstances combine as aids to the success of our operation; while, in addition, "Nature is ever busy, by the silent operations of her own forces, endeavoring to cure diseases," and is continually gathering new energies to resist attacks upon her citadel.

I have intimated that diseased teeth were often as capable of restoration to health as any other part of the system. Nature often recovers of herself, after the severest inflammatory attacks upon the nervous organization, and peridental membranes of the teeth, the result of accident or disease. Depletion in various ways, with treatment of the general system, naturally suggests itself as a remedy for excessive vascular action and congestion, to which the dental organs are liable. When the physical system receives violence from whatever cause, a certain degree of inflammation is essential to her recovery; it is only the *excess* of inflammatory action which produces disorganizing results. It is the province of art to graduate this internal energy of the system, so that it may be expanded at that point of physical restoration for which it was aroused.

In case a tooth, whose pulp is slightly protected, has been subjected to inflammation and pain for no considerable length of time, it can often be restored to health, by first removing such parts of the decay as can be done with safety, or causing too much pain, and then treating it with a concentrated solution of camphor and tannin, together with a small quantity of soda, or tannin, soda and creosote, not very concentrated; a little experience will determine which of the two remedies operate the most kindly. In addition to this, should symptoms indicate an inflammatory condition of the system, especially a tendency to the head, gums and teeth, give cathartics, and let the patient diet; a few days will suffice, when generally it will be safe to proceed and fill the tooth as usual.

In the treatment of the class of inflamed and sensitive teeth under consideration, it was my good fortune several years ago to discover and introduce into my dental practice a new and valuable remedy. I refer to the *tannate of lead.* This I have used in different ways, according to convenience or the character of the case. Sometimes I take the simple powder and work it into white wax, until it has taken up all it is capable of doing, and retain its tenacity; a pill of this of sufficient size to fill the cavity will often produce the most satisfactory results. At other times I have made a solution of the tannate of lead, by mixing it with reduced chloroform, and then adding a few grains of soda. At other times when I could not readily obtain the tannate of lead of the dispensatory, I have found a good substitute in the following: Make a solution of tannin and chloroform, into this put a few grains of acetate of lead, together with a few grains of soda.

By applying a few drops of these solutions upon a pledget of cotton and placing it within the cavity of a sensitive tooth, and at the same time treating the system generally, if indications seemed to require it, I have seldom failed to meet with the most gratifying results.

The relative medicinal character of lead, its influence upon inflammatory action, has long been acknowledged, yet I think it has rarely, if ever been resorted to as a remedy for the teeth. It is true, it has been observed for years, that teeth which were too sensitive to admit of a gold filling, could be readily and painlessly filled with lead and tin. I occasionally adopt this course now, and several months afterwards remove the lead and replace it with gold. From these hints I have adopted its use in the form referred to. The character and uses of the other articles in the formula are apparent.

Some eight years ago, by way of experiment, I used arsenic to reduce the inflammation of the dental bone, and destroy its sensitiveness, but without the intention of destroying the pulp. It was my practice to let the arsenic remain not to exceed two or three hours within the tooth; after removing it, I generally found the sensibility of the tooth exceedingly exalted; filling it carefully with cotton dipped in camphor, I let it remain for several days, when I would generally find that all sensitiveness had ceased. I am sorry, however, to add, that, *ultimately*, I found that quite too many of my teeth died—though by no means a majority of them—under circumstances which gave me some unpleasant reflections in regard to the potency of arsenic. Though I abandoned the use of arsenic under circumstances like the foregoing, I had been sufficiently successful, even with this dangerous remedy, to justify me in the conviction that there was a correct, and a practical principle involved in the idea, and that it could be successfully carried out, if I could only find some milder modification of arsenic. In conversation, a few years after, with some of my professional brethren, *cobalt* was mentioned to me as an excellent article for destroying the nerves of teeth. After inquiring into its nature, I at once commenced using it in the manner I had before used arsenic; and after a few months experience with it, found it equal to my highest anticipations. I have used it, heretofore, combined with white wax, in the same manner that I have used the tannate of lead. I think Dr. Arthur's method of combining it with gutta percha would be far preferable. I do not allow the cobalt to remain in the tooth, generally, more than twelve or fifteen hours, no matter how sensitive the bone of the tooth when the cobalt is removed; a few days time will scarcely ever fail to find the sensitiveness entirely removed. Perhaps the reason why the cobalt has been so readily effectual in my practice when combined with wax, is, that after mixing the wax thoroughly with cobalt, just before introducing it into the cavity I dip the side of the pellet I intend shall come in contact with the bottom of the cavity again into the powdered cobalt, so that when pressed to its place, the cobalt comes in actual contact with the bone. Dr. Arthur's method of applying it with a solution of sandarach and alcohol, I should think—indeed, I *do think*, for since reading his article I have tried it repeatedly—would be a very convenient method especially for shallow cavities.

It is now about four years since I have been established in the use of cobalt in my practice, I consider it one of the most valuable of our dental remedies. It is also a safe remedy; if used with ordinary care, together with a common degree of watchfulness and attention afterwards, one scarcely need ever fail of perfect success.

As Dr. Arthur's experience is fully corroborative of my own and as he has expressed himself at length, and most clearly, on the subject, it is not necessary for me to say more. The inflammation of the periosteum of the tooth, incident sometimes to the use of cobalt, will, of itself, almost invariably subside; if it should continue, a little remedial aid will soon restore it to its former condition.

If a tooth has been painful for any great length of time, and inflammation has so far extended as to commence disorganizing influences upon the structure of the nerve, of course there is no remedy but to remove the pulp and fill the dental canal to its extremity.

Since 1845, I have frequently removed gold stoppings from teeth which were filled upon softened bone, as described in this article, teeth that were filled from one to seven years before, and I have invariably found the former softened parts as hard and insensible as the surrounding bone.

It sometimes occurs, that a tooth filled in the manner described, after a few days have elapsed, gives indication of premonitory symptoms of inflammation of the pulp, together with that of the peridental membrane. In these cases, I temper my remedies to the condition of the case, but always recommend treatment of the general system by cathartics, diet and such other influences as will have a tendency to dissipate concentrated inflammatory action. In connection with this, a repeated application of a solution of sugar of lead to the gums, or simply scarifying the gums and about the necks of the teeth, is oftentimes sufficient.

In more decided cases of inflammation of the nerve, powerful counter-irritants applied to the gums on a line with the tooth and particularly opposite its extremity, rarely fails to subdue it. For this purpose, I use cantharides plaster, nitrate of silver, and, latterly, chloride of zinc. In using the cantharides plaster, I take a small piece of kid or chamois leather, and cut out of its centre a longitudinal strip corresponding to the surface I wish to effect with collodeon or liquid cuticle. I paste upon its back another piece of leather of the same size, this gives a longitudinal cavity which I fill with plaster; after drying the mucous surface as dry as possible, I apply this to the gums over the tooth, letting the lip fall over the leather, as adjusted directing the patient to be careful and not disturb its position. I let it remain until a blister has formed; almost invariable and permanent relief ensues, together with the recovery of the nerve to health.

In using the nitrate of silver, after elevating or depressing the lip, as the case may be, I apply the crude stick to the gums, letting it remain upon one part until they are thoroughly cauterized before I remove it to another. This is often an effectual remedy, and has the advantage over the other of being more convenient.

Recently, I have used chloride of zinc, making a paste with equal parts of flour and zinc, I apply in the same manner I use the cantharides. My experience has not been extensive with this article in this connection, but in some instances its influence has seemed almost magical.

In cases like the foregoing, the treatment is recommended upon the presumption that they have received proper attention, and have been taken *in time.* Of course, if all remedies fail, the removal of the pulp is indispensable to the tooth.

I shall venture before leaving this subject, to present a few cases from my "case book," tracing each case as far as my knowledge of it extends.

"May 6, 1845. Plugged with gold, first right superior molar, for Miss M——, aged 16. Cavity large and quite sensitive; over-capped with light yellow, soft, partially decomposed bone, yielding to the touch and exceedingly sensitive to pressure. Removed all I could of it with safety, treated it with camphor, built an arch over soft parts with foil, and completed the operation without pain. Directed the patient to call next day."

"July 7. Miss M—— called to-day; was unexpectedly called out of town the day after her bad tooth was filled, the reason why she did not call as directed. Says her tooth feels perfectly natural, and has given her no pain, aside from being sensitive to cold and warm drinks, but thinks this wearing away."

"Nov. 16, 1850. Miss M——, now Mrs. F——, called to-day; filled several teeth for her; says the tooth in which I sealed up decay, in 1845, is now one of the best teeth she has; feels perfectly natural. For years it has not been sensitive to cold or warm drinks. It gave every indication of full vitality, good color, no appearance of decay; the large and polished surface of the gold stopping is as perfect as when it was first completed. The joints between the gold and tooth surface, as unbroken, impervious and perfect as ever."

"Sept. 19, 1846. To-day plugged a large cavity in front approximal surface of first inferior left molar, for Mrs. G——, aged 26, nerve nearly exposed, only covered by soft discolored bone, yielding and elastic to touch, soft parts very sensitive to pressure, but not sensitive when undisturbed. Dosed it with concentrated camphor for a few hours, then proceeded and filled it as usual: operation perfectly satisfactory in all respects."

"June 25, 1849. Mrs. G—— called at my office to-day, to have several teeth treated and filled. Consented to let me remove the large plugging I inserted in her lower molar tooth in 1846. She states the tooth had never given her the slightest inconvenience. On removing the gold, I found the walls and bottom of the cavity clean and dry—was gratified to find the slightly discolored bone over the nerve very hard, giving off a crepitus, and ringing as a cutting instrument passed over its surface; it was insensible to pain even when pressed severely with a blunt instrument. The soft parts have evidently received back their quantum of lime; the thickness of the plate of bone between the cavity and the nerve has manifestly greatly increased. Re-plugged the tooth without inconvenience."

"December 20, 1849. To-day removed 21 teeth and roots for Mrs. H., aged 32. In 1844, filled second left inferior molar on grinding surface, cavity very large, excavated nearly all of the bone within the crown; parts just over the nerve, soft, yielding, discolored and very sensitive." Treated and plugged as above. "Removed this one among the rest, though it was entirely free from decay, and the plugging was as bright and firm as ever, for the reason that all the rest of the teeth are so much decayed and diseased they are past remedy. She desires a full set, and under the circumstances I feel justified in removing this with the rest. Held a *post mortem* over the victim tooth. With a watch-spring saw, I cut the tooth in twain from grinding surface, through gold stopping, nerve and roots to apex. The appearance of the exposed sectional surfaces of the different internal parts of the tooth was quite interesting. The nerve was living and healthy. The gold stopping was all that I could desire and had accomplished all that could be expected.

But what was most satisfactory was the appearance of the plate of bone between the gold and nerve; here was two layers or strata of bone, that next to the gold of a yellowish cast, corresponding to parts which in 1844 were "soft, yielding, discolored and very sensitive," but now hard and strong; between this and the nerve cavity, was a layer of bone of pearly whiteness. This last, in my opinion, has been deposited or secreted during the last five years.

"February 24, 1852. Mr. C——, aged 38, called to-day, brought a vial to me containing a tooth which had been lost by a kick from a horse. I find it to be a right superior cuspid, one which I treated in 1847, by filling over decomposed bone overlaying nerve. I recollect the tooth, after being filled, required considerable treatment in consequence of inflammation supervening. On cracking open the tooth, found the nerve was evidently living at the time of the accident, but that the pulp cavity—the *head* of the nerve—was nearly filled with moveable osseous granules, probably induced by inflammation after the tooth was filled. The gold stopping in the tooth was every way satisfactory."

I will give one more case, more particularly to exemplify a hint I have given in another part of this article. In 1844 and '45, I tried many experiments with an amalgam of mercury and silver. A friend of mine called one day to have a molar tooth much decayed, extracted. I induced him to let me try the effect of an amalgam filling in it, for the purpose of watching its influence, &c., &c. The tooth had nothing but the shell of the crown left, and was quite sensitive; but after much patience and more time, I succeeded in removing nearly all of the decay from the walls of the tooth, and a part from the bottom of the cavity. I filled it as well as I could with amalgam with the intention of removing it in a few weeks, even though the pain ceased, for the purpose of noting the effects of the article, which of course I did not regard with much favor.—Next day I saw my patient, who informed me that the tooth had given him no inconvenience. A few weeks afterwards he went to Havana, thence to Europe. I did not see him again until his return in 1848; was surprised to learn from him that he still retained the amalgam tooth, though he said it had become loose within a few months, it had probably ulcerated, though he did not know it. I removed the tooth, found the nerve dead; but on removing the amalgam, I was surprised to find the decay had not advanced since it was filled; and still more surprised to find the soft parts which I had left in the tooth, though quite dark, were completely *fossilized*, being exceedingly hard—even flinty, and when cut, leaving a bright, glittering surface. I could not satisfy myself that the pulp cavity had diminished since the operation.

Since then, I have made it a practice to particularly examine amalgam treated teeth, and have often been surprised to find parts that were evidently once soft, decay, and that about the *walls*, even of the tooth, had become *fossilized*, as though the soft parts had absorbed a portion of the black metallic oxide thrown off from the mercury and silver, so as to arrest decay—as though in fact, a kind of embalming process had transpired! Query—May not a useful hint be gathered from this? may not the dead bone, that intermediate layer in a carious tooth, which must ever over-lie and blend into the living bone, may not this be fossilized by external application of harmless substances?—I am no advocate for amalgam, but am willing to receive a good hint from any source, and shall pursue this subject farther some time.

